# Dupilumab as an innovative therapy for symptomatic management of chemotherapy‐induced pruritus: A case series

**DOI:** 10.1002/ski2.365

**Published:** 2024-03-11

**Authors:** Nupur Singh, Anna Conner, Zachary Nahmias

**Affiliations:** ^1^ University of Tennessee Health Science Center College of Medicine Memphis Tennessee USA; ^2^ Samaritan Dermatology Watertown New York USA

Dear Editor,

Dupilumab is a monoclonal antibody antagonising the interleukin‐4 (IL‐4) receptor, inhibiting the signalling pathways of IL‐4 and IL‐13 and reducing the Th2 response.^1^ Currently, dupilumab has been approved for treatment for atopic dermatitis and prurigo nodularis, and is extensively being studied for use in other dermatologic conditions. As the global burden of atopic dermatitis and other allergic disorders continues to increase, greater research efforts into all treatments' potential uses should be explored.^2^ Other conditions involving pruritus that have had positive responses to dupilumab treatment include chronic idiopathic pruritus and chronic prurigo.^3^ Adverse events reported with dupilumab include injection site reactions alongside enthesis, ophthalmic complications, and head and neck dermatitis among others.^4^

The ongoing other potential uses of dupilumab prompted an investigation of dupilumab use in the management of immunotherapy and chemotherapy‐induced pruritus. [Correction added on 15‐March‐2024, after first online publication: Immunotherapy has been added to preceding sentence.] The pathogenesis of chemotherapy‐induced pruritus has not been fully established and proposed mechanisms may involve unmyelinated C fibres and serotonin, neurokinin 1, opioid, and gamma‐aminobutyric acid receptor activation.^5^ Emerging research is also examining a possible role of the microbiome in influencing chemotherapy‐associated pruritic flares.^6^ Mainstay management of chemotherapy‐induced pruritus includes corticosteroids, antihistamines, and neuromodulators like gabapentin. Other management options include immunomodulatory treatments such as azathioprine or methotrexate.^7^ Still, chemotherapy‐induced pruritus does not have mainstream efficacious treatment, prompting the need for additional treatment options.

We report the notable response to dupilumab treatment in 6 deidentified patients with chemotherapy‐induced pruritus from a rural dermatology hospital practice in the Northeastern United States. Patients were eligible for inclusion if they had either previously been on immunotherapeutic or chemotherapeutic drugs, or were currently undergoing chemotherapy treatment and received oncology clearance. [Correction added on 15‐March‐2024, after first online publication: immunotherapeutic has been added to preceding sentence.] Three patients had finished chemotherapy treatment and were in remission with no evidence of cancer on scans and presented with remaining chemotherapy‐induced itch that developed during their course of treatment. Three other patients were actively on a chemotherapy regimen and had developed chemotherapy‐induced itch. These patients had failed other therapies, which prompted an elected, experimental dupilumab treatment with a follow‐up period of 6 months.

All patients received the standard dosing regimen of dupilumab: an initial 600 mg subcutaneous dose followed by one 300 mg dose every 2 weeks. Coverage for dupilumab was obtained through samples. The average age was 71 years (median 72.5 years [range 58–77 years]; Table 1). Patients' cancers included adenocarcinomas of the lung (2/6), colon (2/6), and breast (2/6). In order to measure response from dupilumab therapy, a percentage response was estimated through patient‐physician clinical discretion at follow‐up visits with the same dermatologist (Table 1). Items measured by the Perceived Stress Score and Dermatology Life Quality Index were used to guide clinical interviewing. Complete response (CR) was indicated for 75% or greater control of itch, partial response (PR) was for 25%–74.99% control, and no response was indicated for less than 25% control or improvement.

**TABLE 1:** Patient demographics and responses to dupilumab therapy.

Patient	Age (years)	Sex (M or F)	Type of cancer	Currently undergoing chemo treatment? (Y or N)	Total number of clinic visits	Response to dupilumab (complete, partial, limited, no)	Patient estimated percent response (%)
1	75	M	Lung	N	2	Complete	90
2	70	F	Colon	N	2	Complete	80
3	68	M	Colon	N	3	Complete	80
4	78	F	Lung	Y	2	Partial	40
5	77	F	Breast	Y	2	Partial	30
6	58	F	Breast	Y	3	Partial	30

Of these 6 patients, 50% (3/6) reported CR above 75%, and the other 50% (3/6) reported PR of at least 25%. Average response was 58.3% with median response at 60%. When further stratified by whether currently undergoing treatment, the average response for patients who had completed treatment was 83.3%. Meanwhile, the average response for patients currently undergoing chemo treatment was 33.3%. No adverse events or reactions were documented or reported.

Potential positive responses of dupilumab therapy in chemotherapy‐induced itch can be attributed to the increase of cytokines and systemic inflammatory responses while a patient is on a chemotherapeutic regimen.^8^ While the working hypothesis is that the itch is due to chemotherapy, IL‐4 and IL‐13 are being extensively investigated for their role in cancer pathophysiology and as potential targets for therapy. All patients had a diagnosis of stage 3 or 4 cancer and were on the standard chemotherapeutic regimens for their respective cancers. Lung adenocarcinoma patients were treated with immunotherapy pembrolizumab (brand name *Keytruda*). [Correction added on 15‐March‐2024, after first online publication: Immunotherapy has been added to preceding sentence.] Colon adenocarcinoma patients received Fluorouracil (brand name *Efudex*). The breast carcinoma patients had triple positive breast cancers (ER+, PR+, HER2+) and were treated with the standard protocol of trastuzumab and tamoxifen. Each of these chemotherapeutic drugs has been linked to drug‐induced pruritus which can remain even after discontinuation of the drug.^9^

Limitations include a small sample size, no standardized assessment tool to survey response, and no control group. Another limitation includes that no long‐term follow‐up was established to observe rate of relapse. Future basic science studies should investigate the relationship of IL‐4 and ‐13 within the pathogenesis of chemotherapy‐induced pruritus. Further clinical research should investigate specific chemotherapeutic regimens that could potentially be more sensitive to dupilumab therapy in order to improve the quality of life for patients undergoing chemotherapy. Overall, our six patients elucidated positive responses to dupilumab while allowing the continuation of necessary immunotherapeutic and chemotherapeutic treatment. [Correction added to 15‐March‐2024, after first online publication: Immunotherapeutic has been added to preceding sentence.] This suggests that dupilumab needs further research and may be an additional, well‐tolerated treatment option to improve control of immunotherapy‐induced and chemotherapy‐induced itch. [Correction added on 15‐March‐2024, after first online publication: immunotherapy‐induced has been added to preceding sentence.]

## CONFLICT OF INTEREST STATEMENT

The authors declare no conflicts of interest.

## AUTHOR CONTRIBUTIONS

**Nupur Singh**: Formal analysis (equal); project administration (equal); visualization (equal); writing—original draft (equal); writing—review and editing (equal). **Anna Conner**: Writing—review and editing (equal). **Zachary Nahmias**: Conceptualization (equal); supervision (equal); writing—review and editing (equal).

## FUNDING INFORMATION

This article received no specific grant from any funding agency in the public, commercial, or not‐for‐profit sectors.

## ETHICS STATEMENT

Not applicable.

## Data Availability

Data sharing not applicable—no new data generated, or the article describes entirely theoretical research.
